# Performance Prediction of Hybrid Bamboo-Reinforced Concrete Beams Using Gene Expression Programming for Sustainable Construction

**DOI:** 10.3390/ma16206788

**Published:** 2023-10-20

**Authors:** Hafiz Ahmed Waqas, Alireza Bahrami, Mehran Sahil, Adil Poshad Khan, Ali Ejaz, Taimoor Shafique, Zain Tariq, Sajeel Ahmad, Yasin Onuralp Özkılıç

**Affiliations:** 1 Department of Civil Engineering, Ghulam Ishaq Khan Institute of Engineering Sciences and Technology, Topi, Swabi 23640, Pakistan; 2 Department of Building Engineering, Energy Systems and Sustainability Science, Faculty of Engineering and Sustainable Development, University of Gävle, 801 76 Gävle, Sweden; 3 Structural Engineering Department, National Institute of Transportation, National University of Science and Technology, Risalpur 23200, Pakistan; 4 Department of Civil Engineering, Necmettin Erbakan University, 42090 Konya, Turkey; 5 Department of Civil Engineering, Lebanese American University, Byblos 1102-2801, Lebanon

**Keywords:** green building material, hybrid beams, bamboo-reinforced concrete beam, finite element model, replacement, gene expression programming

## Abstract

The building and construction industry’s demand for steel reinforcement bars has increased with the rapid growth and development in the world. However, steel production contributes to harmful waste and emissions that cause environmental pollution and climate change-related problems. In light of sustainable construction practices, bamboo, a readily accessible and eco-friendly building material, is suggested as a viable replacement for steel rebars. Its cost-effectiveness, environmental sustainability, and considerable tensile strength make it a promising option. In this research, hybrid beams underwent analysis through the use of thoroughly validated finite element models (FEMs), wherein the replacement of steel rebars with bamboo was explored as an alternative reinforcement material. The standard-size beams were subjected to three-point loading using FEMs to study parameters such as the load–deflection response, energy absorption, maximum capacity, and failure patterns. Then, gene expression programming was integrated to aid in developing a more straightforward equation for predicting the flexural strength of bamboo-reinforced concrete beams. The results of this study support the conclusion that the replacement of a portion of flexural steel with bamboo in reinforced concrete beams does not have a detrimental impact on the overall load-bearing capacity and energy absorption of the structure. Furthermore, it may offer a cost-effective and feasible alternative.

## 1. Introduction

The production of building materials, including concrete and steel, has contributed to the rise in environmental pollution [1,2]. The construction sector is acknowledged as one of the most significant contributors to environmental pollution on a global scale [3]. The production of steel and concrete has a negative environmental impact [4,5,6]. In order to mitigate the carbon footprint associated with construction materials, it is crucial to allocate resources toward sustainable alternatives [7] that can meet the increasing demands of the building and construction industry.

Bamboo, a forest product with significant social, economic, and ecological importance, has emerged as a promising natural and sustainable resource capable of substituting for traditional construction materials [8]. Its growing popularity signifies its potential as an alternative to replace steel reinforcement in reinforced concrete structures [9]. There has been a growing adoption of bamboo as a building material, primarily thanks to its environmentally friendly characteristics [10]. Bamboo’s rapid renewability, with a faster growth rate compared to other trees, makes it highly suitable as a sustainable source of wood for various industries, particularly in construction projects [11]. Its promising features make it appropriate to be used as a substitute for structural wood in buildings [12], thereby aiding in the preservation of the environment worldwide.

Limited literature is available on bamboo as a reinforcement material compared to steel reinforcement. Beams, columns, and slabs, some of the main elements of bamboo-reinforced concrete (BRC), have been tested in the past, and the results have provided a critical evaluation of the applicability of BRC. Researchers have compared the material advantages and disadvantages of BRC in contrast to steel-reinforced concrete (SRC). They have highlighted aspects such as durability, mechanical properties, and environmental friendliness to better understand their respective attributes [13].

Numerous researchers have conducted studies on the material characteristics of bamboo. Consistently, it has been noted that bamboo exhibits a slightly greater tensile strength when compared to its compressive strength [14]. Bamboo material typically reaches its peak strength between the ages of three and five years, with the weakest point in a bamboo culm being identified as a knot under tensile loading [15]. Takeuchi et al. [16] conducted experimental research to evaluate Young’s modulus and Poisson’s ratio of bamboo. The study’s outcomes revealed that the material’s mechanical properties were influenced by its physical anisotropy, indicating variations in different directions of the material. Studies have reported that impermeable bamboo reinforcement requirements can be met by a wide variety of waterproofing materials. Moreover, material decay, swelling, etc., should be controlled through the application of available compounds [17]. Bamboo is characterized by its remarkable flexural ductility, which is the result of the interaction between its tough exterior and compressible interior [18]. This combination enables the material to withstand tensile stress while undergoing significant flexural deformation.

In a few studies, the performance of bamboo-reinforced structures was tested under different types of loadings. Dewi and Nuralinah [19] performed a study to evaluate the effects of incorporating bamboo reinforcements, specifically pegs, in concrete beams subjected to a four-point loading configuration. Their research results demonstrated that the inclusion of these pegs, in conjunction with bamboo reinforcement, led to an augmentation in both the capacity and strain energy of the concrete beams. According to Tan et al. [20], a concrete beam reinforced entirely with bamboo was observed to attain roughly 46% of the load-bearing capacity of a comparable concrete beam reinforced with steel. Ramaswamy [21] carried out an investigation focusing on the maximum deflection and load-bearing capacity during a three-point bending test involving various types of beams. These included a plain concrete beam, a singly reinforced BRC beam, and a doubly reinforced BRC beam. The results provided a substantial increase in the load-bearing capacity of the singly and doubly reinforced BRC beams, approximately 200% and 250% higher, respectively, when compared to the plain concrete beams. The research work done by Khan [22] unveiled that the geometry of bamboo reinforcement played a substantial role in influencing the performance of BRC beams. Square-shaped BRC beams had superior flexural strength in comparison to triangular and rectangular BRC beams.

In a recent study, bamboo underwent a series of mechanical tests aimed at determining its properties. These tests included tension, bond strength, and water absorption assessments, which underscored the noteworthy influence of knots on bamboo’s tensile strength. The measured tensile strength of bamboo was determined to be 101 MPa, roughly equivalent to 25% of grade 60 steel, while its Young’s modulus was found to be 19,505 MPa, representing about 10% of the value of steel. Furthermore, the study reported that BRC beams with corrugation displayed an increased ultimate capacity compared to regular BRC beams. Among the various bamboo types, corrugated bamboo exhibited the most beneficial effect in preventing the bond slip and providing sufficient flexural capacity. Notably, all the beams experienced failure due to pure flexure, with cracking originating in the pure bending zone. It was concluded that utilizing corrugated bamboo could be advantageous in enhancing the flexural capacity and preventing the bond slip in BRC beams [23].

Moreover, there has been a noticeable surge in the adoption of soft computing techniques for constructing empirical models. Recently, researchers have shown keen interest in employing gene expression programming (GEP), a popular soft computing method, in various engineering domains to develop predictive models [24,25]. Several GEP models were created in [26,27] to predict the shear strength of both exterior and interior reinforced concrete beam-to-column joints under monotonic and uniaxial loading conditions. These GEP models exhibited exceptional performance when exposed to biaxial cyclic loading, surpassing code-based formulations in terms of accuracy and reliability. Numerous studies [28,29,30,31,32,33,34,35,36,37,38,39,40,41,42] have demonstrated the utility of GEP as an effective technique to develop predicting models in various civil engineering applications. In recent times, the research landscape has revealed a noticeable absence of substantial endeavors in predicting or establishing a GEP-based model for evaluating the flexural strength of BRC beams. In this context, the application of machine learning techniques [43,44] has emerged as a prominent contender, offering the ability to predict uncertainty in material properties and configurations with a high level of accuracy. Various evolutionary techniques have been employed to develop equations for squat wall shear [45]. Among the artificial intelligence-based methods, GEP stands out owing to its distinctive chromosome representation, setting it apart from other known techniques [46]. In summary, there is a pressing requirement to reassess the flexural strength model for BRC beams by compiling existing experimental data. This motivation has propelled the current study to develop a reliable and user-friendly predictive model using GEP. The application of this model is anticipated to result in substantial cost and time savings.

From the literature review, it has become evident that the majority of research efforts have been directed toward bamboo treatment, the mechanical properties of bamboo, and the fire resistance of BRC [14,47]. Nevertheless, there is a research gap concerning the comparative study of the flexural strength between hybrid BRC beams and SRC beams. While recent studies have mainly emphasized experimental investigations, there remains a significant gap in numerical investigations related to the performance of BRC beams. The concept of hybrid BRC beams has yet to be fully explored, as it could potentially offer the strength comparable to SRC beams without considerably compromising the performance. Therefore, this study aims to assess the viability of employing bamboo as a replacement material for steel in reinforced concrete structures. Additionally, this research attempts to conduct numerical simulations of full-size BRC beams, as past research has predominantly centered on small-scale experiments. The finite element models (FEMs) for both BRC and SRC beams were carefully developed, considering the nonlinear characteristics of concrete, steel, and bamboo. Subsequently, the specimens were subjected to testing under three-point loading conditions. Afterward, GEP was employed to propose a strength assessment model for hybrid BRC beams. A comprehensive explanation of the numerical modeling, the implementation of GEP, and the results and discussion of the analyses are presented in the following.

## 2. Materials and Methods

### 2.1. Constitutive Modeling of Material

The concrete damaged plasticity (CDP) model is a widely accepted method used for simulating the inelastic behavior of concrete and other materials with quasi-brittle characteristics in the finite element analysis (FEA) [48]. The CDP model is a continuum-based damage model designed for concrete, which integrates plasticity. This model takes into consideration two critical failure mechanisms observed in concrete materials: compressive crushing and tensile cracking. To regulate the evolution of the yield surface, two hardening factors, $\varepsilon_{c}^{pl}$ and $\varepsilon_{t}^{pl}$, are employed. These factors represent the compressive and tensile equivalent plastic strains, respectively, corresponding to the failure mechanisms under compressive and tensile loadings. These damage variables can vary from 0 to 1, with 0 signifying an undamaged material and 1 indicating a complete loss of strength. The stress–strain relationships under uniaxial compressive and tensile loadings are described by the equations below.

For tensile loading:

(1) $$\sigma_{t}=\left(1-d_{t}\right)E_{o}(\varepsilon_{t}-\varepsilon_{t}^{pl})$$

For compressive loading:

(2) $$\sigma_{c}=\left(1-d_{c}\right)E_{o}(\varepsilon_{c}-\varepsilon_{c}^{pl})$$

where $E_{o}$ represents Young’s modulus of the material, and $d_{t}$ and $d_{c}$ designate the tensile damage variable and compressive damage variable, respectively. The plastic flow parameters for the concrete material were adopted from previous research works [49,50,51].

**Figure 1:**
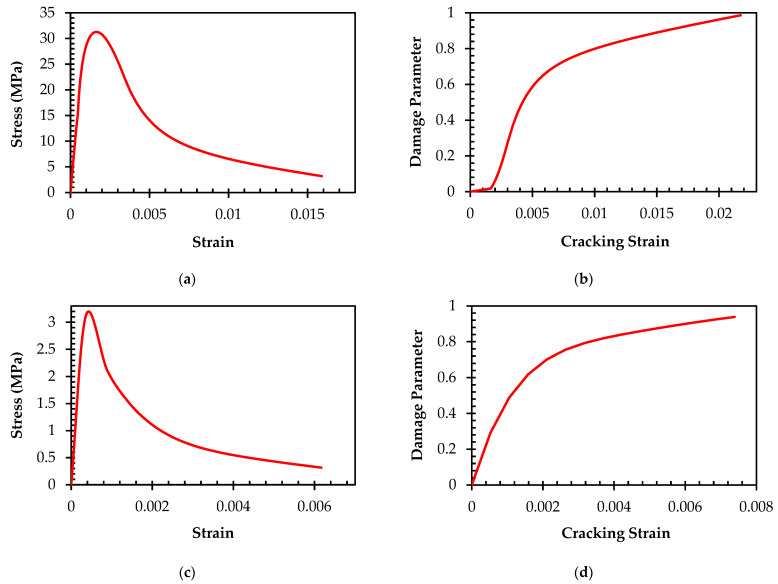
(**a**) Concrete compressive stress–strain curve; (**b**) concrete compressive damage; (**c**) concrete tensile stress–strain curve; (**d**) concrete tensile damage.

The concrete behavior in uniaxial stress–strain is typically divided into three distinct stages: hardening, linear-elastic, and post-peak softening. The linear-elastic stage corresponds to the initial loading up to the elastic limit (12.38 MPa), as depicted in Figure 1a. The hardening stage of the stress–strain curve is defined by the ascending portion of the curve, starting from the elastic point (12.38 MPa) and extending up to the peak stress (31.31 MPa). Following the peak stress, the post-peak softening stage corresponds to the initiation and progression of compressive damage in the concrete material until it reaches the ultimate compressive strain. This characterization of the concrete behavior is important for understanding the failure mechanisms and predicting loads of failure for concrete structures. In the CDP model, the initiation of damage in uniaxial compression is defined during the softening procedure, which begins at the peak compressive strength. As the cracking strain increases, the damage increases in a nonlinear manner, as illustrated in Figure 1b. This comprehension of the connection between cracking strain and damage is essential for a deeper understanding of concrete failure mechanisms and is invaluable in the analysis and design of concrete structures.

In the context of tensile loading, the uniaxial stress–strain behavior of concrete is simulated through a two-phase approach, as shown in Figure 1c. The first phase describes the linear elastic behavior of concrete until its tensile strength is reached. The second phase, which is characterized by the beginning and propagation of cracks in concrete, results in a nonlinear stress–strain relationship. This is important for improving the analysis and design of concrete structures, as it allows for a more near-true prediction of the performance of concrete under tensile loading. The initiation of damage in uniaxial tension within the CDP model is defined at the point of tensile strength, as displayed in Figure 1d.

**Figure 2:**
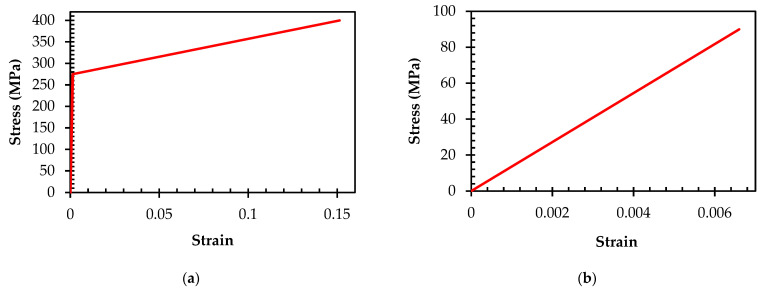
Typical stress–strain behavior introduced to numerical model: (**a**) steel; (**b**) bamboo.

The behavior of steel was simulated using an elastic–plastic modeling approach [52,53]. The properties of concrete, steel, and bamboo utilized in this study were obtained from previous research studies [8,23]. The material behavior of steel reinforcement is characterized by both elastic and plastic behaviors, with the elastic behavior described by Young’s modulus and the plastic behavior defined by the post-yielding Young’s modulus. The characteristics of the plastic phase are represented by bilinear behavior, resembling the typical stress–strain relationship of reinforcement that is integrated into the model, as presented in Figure 2a. The mechanical response of bamboo reinforcement encompasses the linear response until its failure. Young’s modulus quantifies the linear behavior, while the failure behavior is delineated by the average strength, as noted by Qaiser et al. [23]. Figure 2b provides a visual representation of the stress–strain relationship observed in bamboo testing.

Table 1 gives an overview of the properties and attributes of bamboo and steel reinforcement employed in the study, while Table 2 outlines the parameters utilized to model the concrete behavior using the CDP approach [54].

### 2.2. Description of Model

The behaviors of BRC and SRC beams were modeled using ABAQUS/CAE software [56]. A typical concrete beam had the cross-sectional dimension of 500 × 500 mm and the length of 4300 mm. It was designed with a 50 mm clear cover on each side of the beam. Shear stirrups, 10 mm in size, were spaced 200 mm apart. The initial dimensions for the concrete beams were primarily influenced by the existing experimental data found in the literature, with square cross-sections being the predominant configuration [23]. However, it is important to clarify that the analysis was not limited exclusively to square cross-sections. Instead, a comprehensive parametric study encompassed a diverse range of beam depths and incorporated rectangular cross-sections. This deliberate approach aimed to ensure the broad applicability and versatility of the analysis, accommodating various beam dimensions.

**Figure 3:**
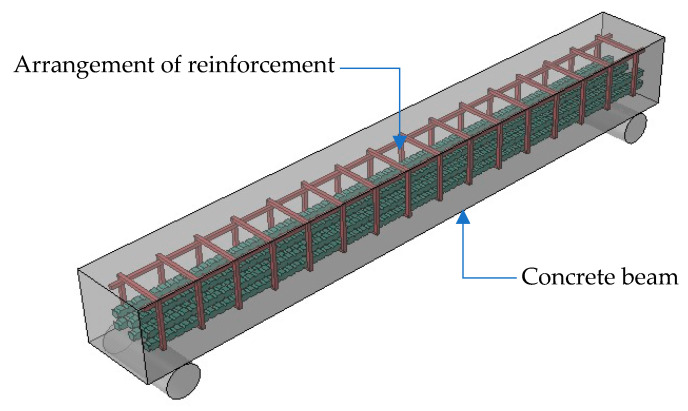
Typical concrete beam specimen with different reinforcement configurations, as detailed in Table 3.

Given the scarcity of research regarding comparative studies on the flexural strength between BRC and SRC beams, the study involved the modeling of five beams, as detailed in Table 3, each with distinct reinforcement configurations. A typical concrete beam specimen with different reinforcement configurations, based on Table 3, is depicted in Figure 3. In Table 3, model B1-4SB-SS was used as the control beam, which consisted of steel reinforcements and steel stirrups. Model B2-4BB featured bamboo bars as longitudinal reinforcements without stirrups. In model B3-4BB-BS, bamboo stirrups were utilized to hold bamboo bars. Model B4-4BB-SS replaced bamboo stirrups with steel stirrups while keeping reinforcements as the same as model B3-4BB-BS. Lastly, in model B5-2BB-2SB-SS, the beam was reinforced with 50% steel bars, 50% bamboo bars, and steel stirrups, and it was referenced as the hybrid beam.

**Figure 4:**
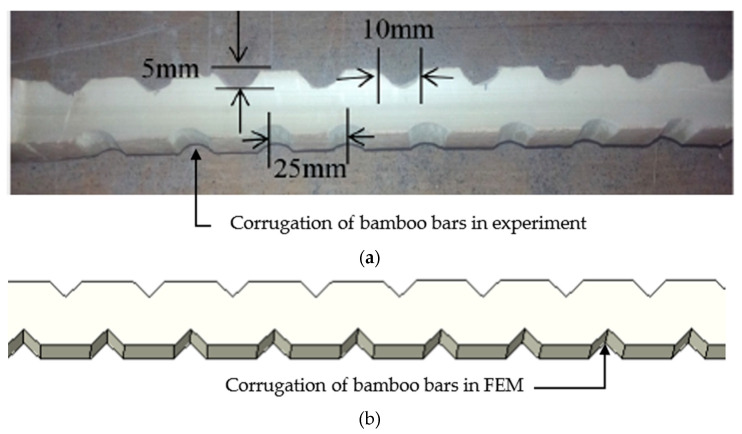
Geometry of corrugated bamboo reinforcement: (**a**) experiment [23]; (**b**) FEM.

Five beam models were created with varying reinforcement distributions. The reinforcement for each beam was determined based on the control beam, which was a typical beam with steel rebars only. The minimum area of steel was calculated for each beam, and the number of bars was decided accordingly. The minimum area of steel was calculated using the following formula [57].

(3) $$A_{s,\;min}=\frac{1.4}{f_{y}}b_{w}d$$

where *b_w_* and *d* are the width and effective depth of beam, respectively. A 22 mm diameter steel bar was employed to provide the required steel reinforcement, and the same number of bars was provided in all the specimens. For the analysis of the BRC beams, corrugated bamboo with the size of 20 × 20 mm was utilized, as illustrated in Figure 4. This modification served to prevent the bond slip and enhance the overall performance, in line with past research practices [23].

### 2.3. Boundary Conditions, Interactions, and Loading

**Figure 5:**
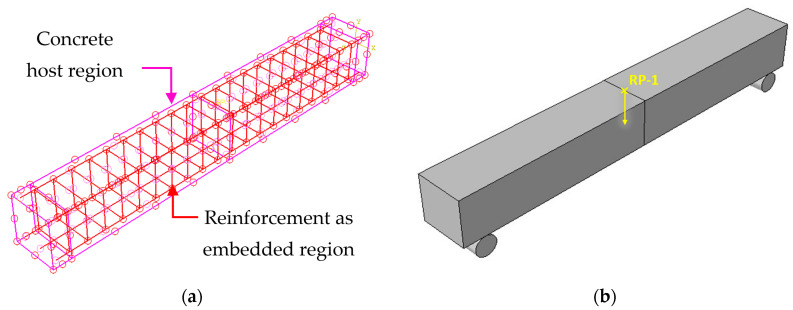
(**a**) Embedded reinforcement and host beam; (**b**) boundary conditions and load application.

In this study, it was assumed that there was a strong bond between bamboo reinforcement facilitated by the corrugated nature of bamboo. As a result, the embedded region method was employed to simulate the bond between concrete and reinforcement. This modeling technique entails evaluating the stiffness of the reinforcement elements separately from the concrete elements. It effectively addresses the challenges associated with mesh limitations that are often encountered in discrete reinforcement modeling approaches. By using this technique, the host element (concrete) and the slave element (bamboo/steel) were seamlessly connected, as shown in Figure 5a. Further, this approach guarantees that the displacement of the surrounding concrete components is compatible with the displacement of the reinforcement elements, resulting in a more accurate representation of the bond between the two materials.

To obtain the load–deflection curve, a displacement-controlled 10 mm loading was applied at the reference position (RP-1). The kinematic coupling constraint was used to apply the load through the reference node and coupling nodes. The reference point and the top surface were interconnected through a kinematic link. Following this process, the load–deflection curve was generated at the reference position. To replicate the boundary conditions observed in the experimental work, roller supports were employed at both ends [23]. The specific details of the boundary conditions and kinematic coupling are shown in Figure 5b.

### 2.4. Meshing

**Figure 6:**
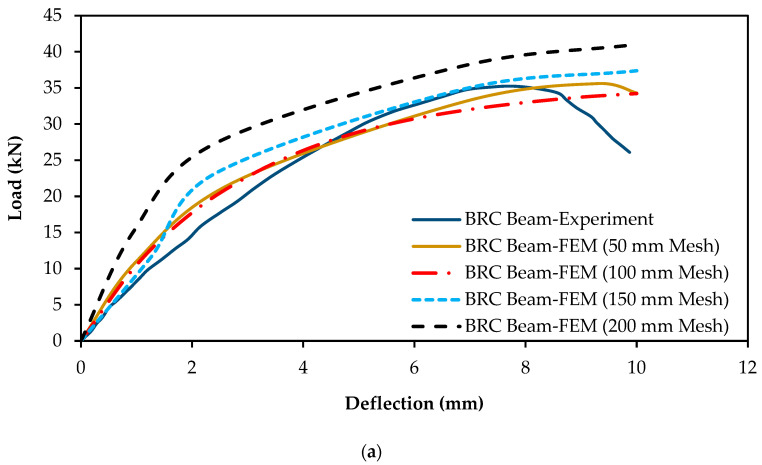

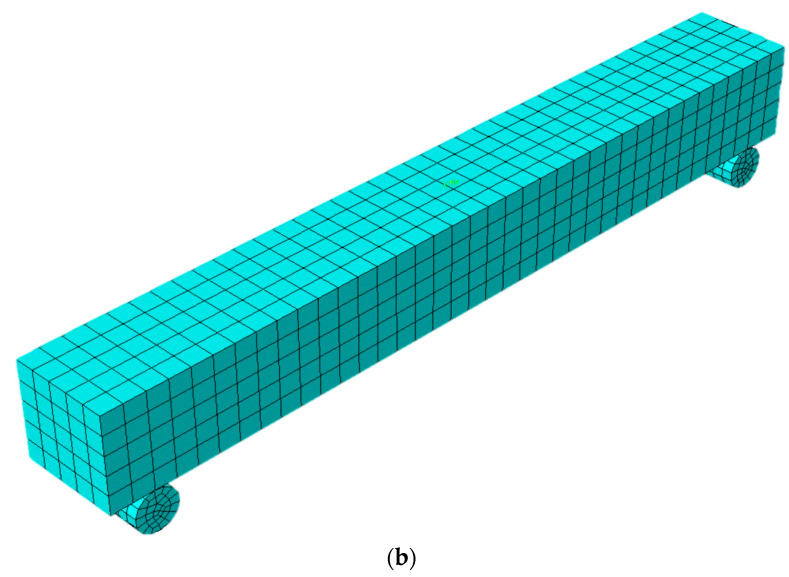
(**a**) Mesh sensitivity analysis of BRC beams; (**b**) meshed geometry of numerical model.

Meshing of solid elements was done using reduced integration with 3-dimensional continuum eight-node elements (C3D8R). For meshing of the bar elements, two-node 3-dimensional truss elements (T3D2) were employed. To assess the mesh sensitivity of the models, an analysis was conducted using different mesh sizes, namely, 200 mm, 150 mm, 100 mm, and 50 mm. The results of the analysis indicated that refining the mesh size beyond 100 mm had only a negligible impact on the predicted outcomes and did not significantly alter the behavior of the model, as displayed in Figure 6a. Thus, the mesh size of 100 mm was considered sufficient to achieve accurate and reliable results for the numerical simulation. The smaller mesh size took more computational time and demonstrated no considerable changes in the numerical results. The meshed geometry of the numerical model is depicted in Figure 6b.

### 2.5. Validation of Model

In a previous study [23], both BRC and SRC beams were experimentally tested. The beams had the cross-sectional dimension of 230 × 230 mm with the length of 1.88 m, and they had a clear cover of 38 mm on each side and the bottom. The reinforcement consisted of two layers of tensile reinforcement and hanger bars to hold the stirrups with the spacing of 115 mm. The experimental setup involved three-point loading with supports placed at 75 mm from both ends. Two numerical models were developed using the experimental data and properties from Table 1 to evaluate the effectiveness of FEM. The main objective of this analysis was to validate the suitability of FEM for predicting the behavior of BRC and SRC beams. This validation process was carried out by comparing the FEA results with the experimental data obtained for the two specific beams under investigation. The objective was to confirm the accuracy and reliability of FEM in replicating the real-world behavior of these reinforced concrete beams.

**Figure 7:**
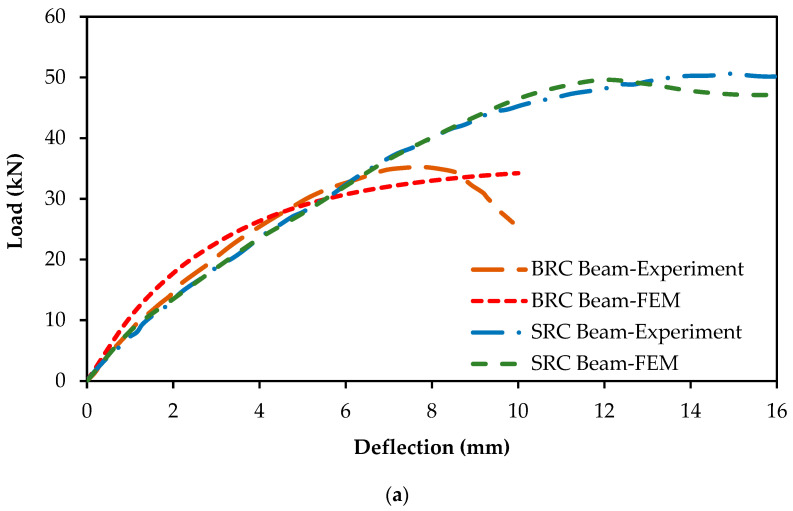

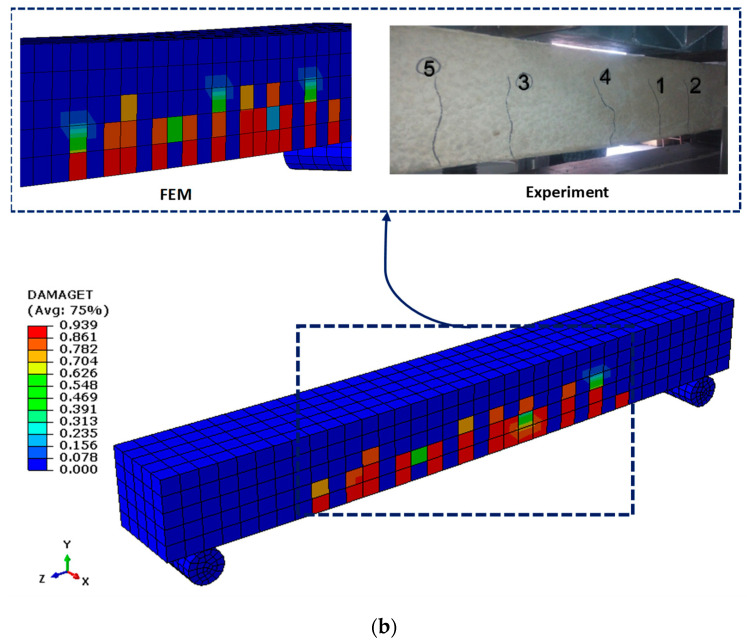
(**a**) Comparison of load–deflection responses of FEM and experimental study [23]; (**b**) comparison of failure patterns between FEM and experimental results [23] for BRC beam.

The numerical modeling of the test specimens was executed by implementing the methods detailed in the preceding sections. These methods were utilized to faithfully represent the constitutive behavior of the materials and incorporate the essential modeling parameters. The load–deflection responses of the specimens obtained from both experiment and FEM analyses were compared, as illustrated in Figure 7a. The findings from the FEM analyses closely aligned with the experimental results. It is crucial to highlight that there was minimal disparity between the load–deflection curves of the SRC beams obtained through experiment and those generated through FEM. Furthermore, the failure load achieved through FEM was approximately 4% higher at the deflection of 12 mm, whereas the experimental failure load was roughly 6.5% higher at the deflection of 15 mm. In both the FEM and experimental results for the SRC beams, a consistent deformation value of 10 mm was observed (Figure 7a). In addition, the performance of both curves was almost indistinguishable until they reached the point of failure, which occurred beyond the 10 mm deformation point.

For the BRC beams, the FEM curve was steeper than the experimental curve, from the starting point to the point of the deflection at 4.3 mm. Meanwhile, the experimental failure load had a 2.85% higher value than the FEM results.

The load–deflection curves derived from both experimental testing and FEM analyses of the SRC and BRC beams exhibited a high degree of similarity, indicating a strong agreement between the experimental and FEM outcomes, as summarized in Table 4.

The analysis of the energy absorption in this study highlighted a strong concordance between the FEM and experimental results. In the case of the SRC beam, there was only a minimal deviation of 0.18% between the FEM result (555.65 kN-mm) and the experimental measurement (556.63 kN-mm). Similarly, for the BRC beam, while the difference between the FEM (252.78 kN-mm) and experimental (243.47 kN-mm) results was slightly higher as 3.82%, it still signified a substantial agreement between the two approaches.

The numerical model also showed a strong correlation between the cracking pattern observed for the BRC beams during the test and the pattern predicted by FEM. Figure 7b displays a fair comparison between the cracking patterns witnessed during the test and those predicted by FEM. These results confirmed the reliability of FEM in accurately capturing the actual behavior of the beams, thereby validating the precision and robustness of numerical methods of this research.

## 3. GEP Algorithm

GEP is a type of genetic algorithm (GA) used to construct mathematical models from input data in a domain-independent manner. Unlike traditional GAs and genetic programming (GP), GEP employs a distinctive chromosome representation. While GAs utilize linear strings of constant length and GP uses various nonlinear entities of varying sizes and shapes [58,59], GEP stands out by combining a fixed-length linear string with a branching structure that can vary in size and shape.

The GEP evolutionary process encompassed numerous iterations, where refinements were applied to various parameters. These adjustments included modifications to factors such as the quantity of chromosomes, genes, head sizes, and linking functions. Through this iterative approach, GEP was able to identify the most promising candidates within the initial population, making selections based on their fitness levels and ultimately optimizing the solutions. It is important to highlight that although increasing the number of genes and chromosomes can lead to complex functions that closely align with the results, a delicate balance needs to be maintained. This equilibrium entails simplifying the mathematical model by regulating the number of genes and chromosomes, all while attaining the desired level of accuracy [58,59,60,61].

Attaining convergence toward the global optimal solution stands as a pivotal element within the GEP algorithm. Nonetheless, scenarios arise where the algorithm encounters challenges in discerning the superior solution amidst multiple competing candidates. The consequence of this situation can be an infinite sequence of steps, potentially resulting in a program that fails to conclude or an expression that lacks logical consistency. To address this issue, adjustments can be made to the linking functions or the number of genes and chromosomes, with the goal of improving the algorithm’s efficiency [62].

In the last decade, the benefits of GEP have contributed noticeably to its increasing prominence within the field of structural engineering. A multitude of scholars [63] have harnessed the power of GEP to construct advanced models that precisely gauge the capacity of diverse structural components. In this investigation, GEP was adeptly harnessed to predict the flexural strength of the hybrid BRC beams.

**Figure 8:**
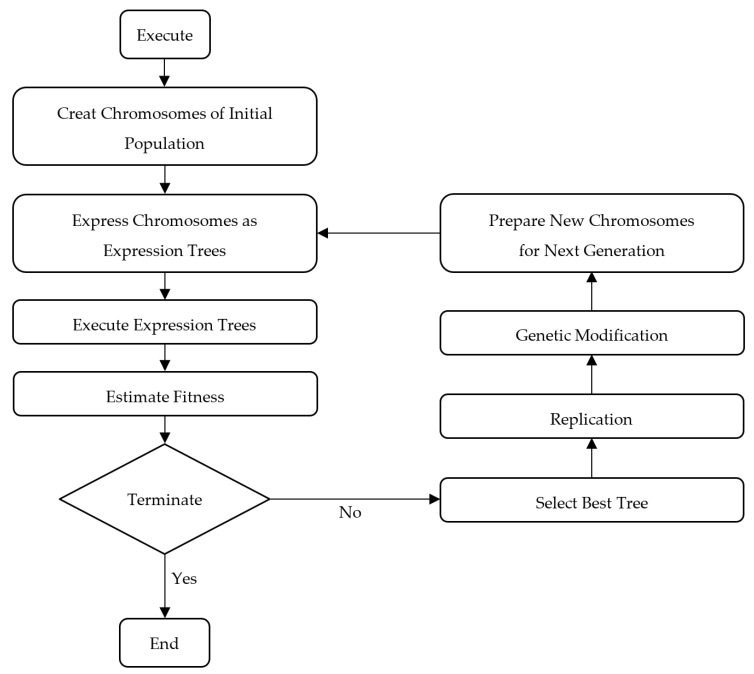
Flow chart representing steps of GEP [64].

Figure 8 provides the multiple stages involved in the optimization process of GEP. This process commences with the selection of control parameters, which encompasses the function set, terminal set, fitness function, control parameters, and stopping conditions. Before the execution of the evolutionary algorithm, the fitness function is defined, and an initial population of random strings, known as “chromosomes” in GP terminology, is generated. These strings are translated into expression trees, and the fitness scores of each chromosome are evaluated based on their outcomes. If the fitness criteria are not met, a roulette-wheel sampling method is employed to select specific chromosomes for mutation, ultimately leading to the creation of new generations. Conversely, when the variables are finely tuned to align with the fitness function, the chromosomes undergo optimization.

### 3.1. Parametric Study

A total of 127 models were created in ABAQUS by changing different key parameters. The dataset used for developing a reliable predictive model included important influencing parameters. Identifying these parameters necessitated a thorough examination of experimental investigations. Critical factors, consisting of the concrete’s cross-sectional area, reinforcement area, beam depth, beam span length, and concrete compressive strength, played a pivotal role. Table 5 gives an overview of these crucial parameters, including their respective ranges within the dataset.

**Figure 9:**
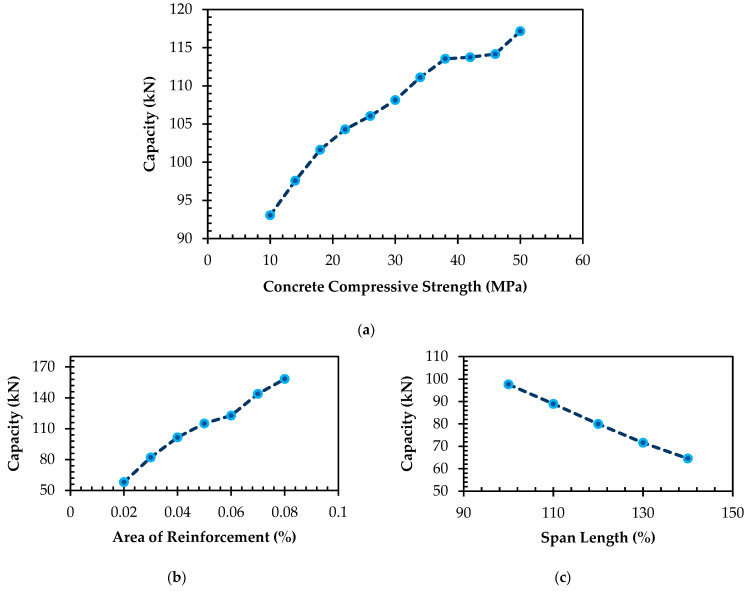

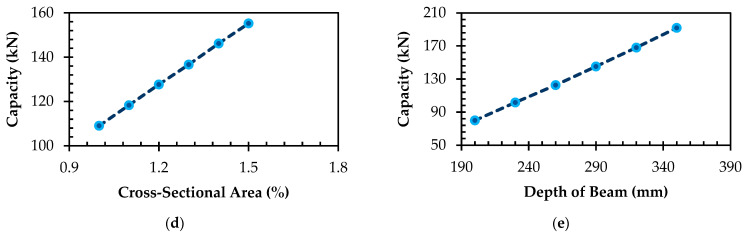
Parametric study: (**a**) capacity vs. *f’_c_*; (**b**) capacity vs. *A_r_*; (**c**) capacity vs. *L*; (**d**) capacity vs. *A_c_*; (**e**) capacity vs. *D*.

In Figure 9a, it is evident that the flexural capacity of a beam experienced enhancement as the concrete compressive strength increased. For instance, a beam with the concrete compressive strength of 10 MPa exhibited the flexural capacity of 93.06 kN, whereas a beam having the concrete compressive strength of 50 MPa demonstrated the higher flexural capacity of 117.16 kN, marking the notable 25.9% increment. This was because stronger concrete could withstand more tensile stress, which was the primary type of stress that was experienced in a beam under flexure.

As depicted in Figure 9b, there has been a clear trend indicating that the flexural capacity of a beam rose in tandem with the augmentation of the area of reinforcements. Specifically, when considering a beam featuring 2% reinforcements, its flexural capacity was 58.06 kN. In contrast, a beam incorporating a more substantial 8% reinforcements showed the considerably higher flexural capacity of 158.45 kN, reflecting the impressive 172.91% enhancement. This phenomenon can be attributed to the reinforcements’ ability to fortify the tensile strength of the beam, thereby contributing to its capacity to withstand the tensile stresses inherent to the flexural loading.

According to Figure 9c, the beam’s capacity showed a declining trend with an increase in the span length. This phenomenon can be attributed to the fact that a beam with an extended span would endure heightened tensile stresses, ultimately leading to a diminished capacity. As an example, the analysis revealed that a beam with the span length of 5 m resulted in the capacity of 100 kN. Conversely, a beam with the identical cross-sectional dimension and reinforcements but featuring the extended span length of 10 m registered the reduced capacity of 75 kN, signifying the noteworthy 25% decrement.

As observed in Figure 9d,e, a discernible linear relationship was established between the flexural capacity of a beam and its respective cross-sectional area and depth.

### 3.2. Proposed GEP Model for Estimating Flexural Strength of Hybrid BRC Beams

**Figure 10:**
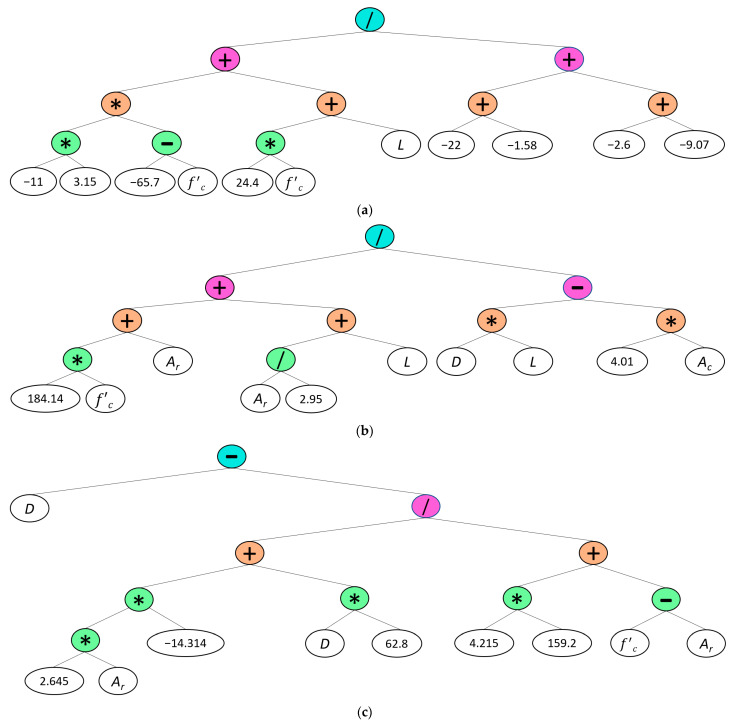
Gene expression tree for calculation of flexural strength: (**a**) sub-ET 1; (**b**) sub-ET 2; (**c**) sub-ET 3.

In this section, a GEP model is developed to predict the flexural capacity of the hybrid BRC beams. The equation employed to represent these GEP models, which was extracted from sub-ET (sub-elemental tail) of GA, was derived from the aforementioned dataset.

(4) $$P_{u}=G_{1}+G_{2}+G_{3}$$

(5) $$G_{1}=-\left(1.63{f\prime }_{c}+0.03L+61.13\right)$$

(6) $$G_{2}=\frac{184.14{f^{\prime }}_{c}+1.34A_{r}+L}{DL-4A_{c}}$$

(7) $$G_{3}=D-\frac{62.8D-37.8A_{r}}{{f^{\prime }}_{c}-{\;A}_{r}+671.9}$$

where *L* and *D* designate the length and depth of the beam, respectively, *f’_c_* represents the concrete compressive strength, and *A_r_* and *A_c_* denote the areas of reinforcements and cross-section, respectively. The graphical representation of the estimation model’s expression tree is also presented in Figure 10 along with the parameters for construction of the model listed in Table 6.

### 3.3. Accuracy and Validation of Proposed GEP Model

The precision and reliability of empirical models are inherently intertwined with the meticulous process of training and validating these models. In this particular study, the model’s development leveraged a training dataset comprising a randomly selected 85%, while a distinct and equally random 15% subset was reserved for validation purposes. After constructing the model, a statistical evaluation of its performance, including metrics like the coefficient of determination (*R*^2^), was employed to quantitatively gauge the model’s effectiveness. *R*^2^, which assesses the reliability of the model, can be calculated using the following equation:

(8) $$R^{2}=1-\frac{{\sum [Experimental\;value-predicted\;value]}^{2}}{{\sum [Experimental\;value-{\;Experimental\;value}_{mean}]}^{2}}$$

**Figure 11:**
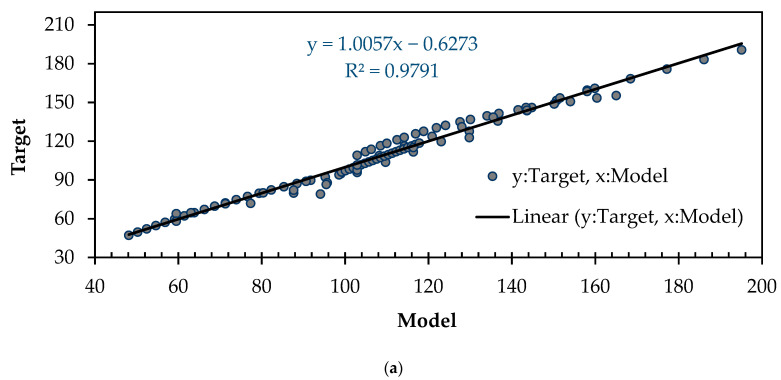

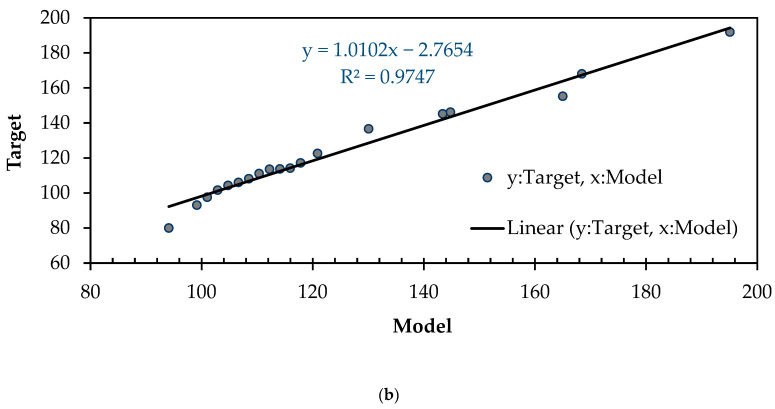
Comparisons of model and target results for flexural strength of hybrid BRC beams: (**a**) training dataset; (**b**) validation dataset.

An *R*^2^ value approaching 1 indicates a precise prediction. The statistical assessment of the model’s performance, in comparison to the numerical results referred to as ‘‘target’’, is illustrated in Figure 11. An examination of *R*^2^ provided a value of 0.98 for the training dataset and 0.97 for the validation dataset.

The successful application of GEP in predicting the flexural strength without the need for numerical or experimental work is a significant finding. *R*^2^, which quantifies the accuracy of the regression analysis, revealed that our results were highly precise. Moreover, the equation derived from the expression tree demonstrated its efficacy in accurately calculating the flexural strength. The similarity between the predicted magnitudes of the flexural strength obtained through GEP and those extracted from FEMs further validated the reliability of our approach. This breakthrough in accurately estimating the flexural strength not only saves time and resources but also offers a promising alternative to traditional experimental methods. These findings open up new avenues for leveraging GEP as a powerful tool for predicting and analyzing complex material properties in various engineering applications.

## 4. Results and Discussion

### Load–Deflection Curves and Energy Absorption of All Models

**Figure 12:**
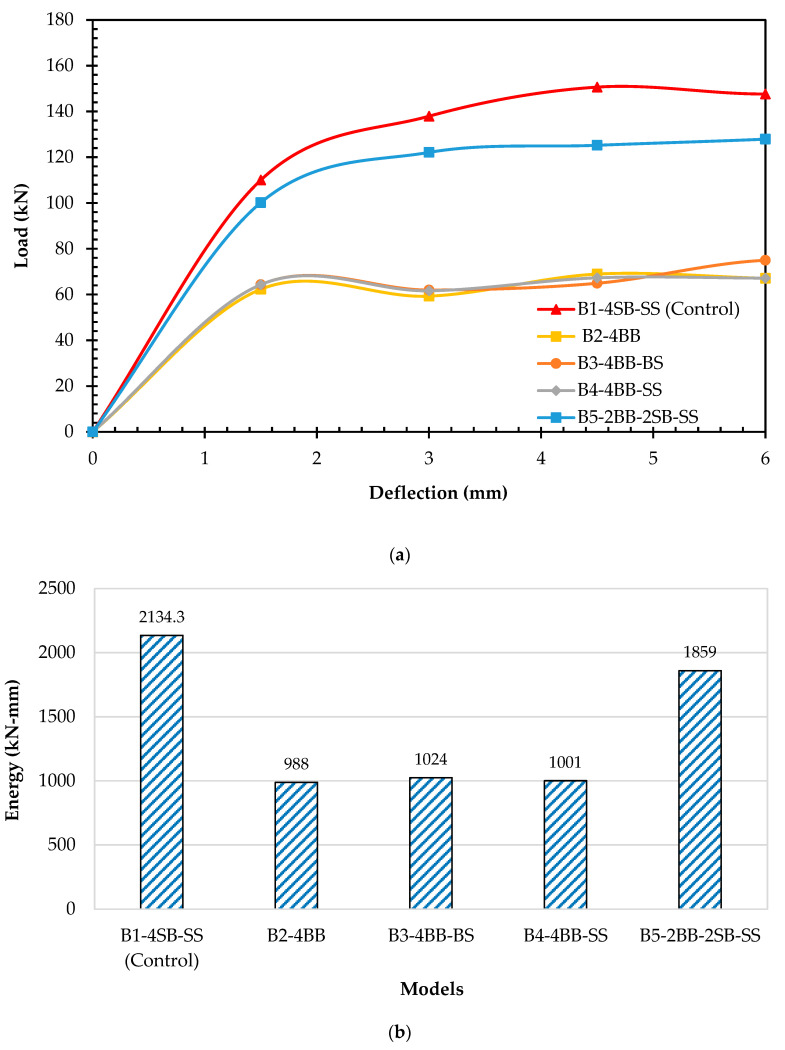
(**a**) Load vs. deflection response of all beams; (**b**) energy absorption of all beams.

**Figure 13:**
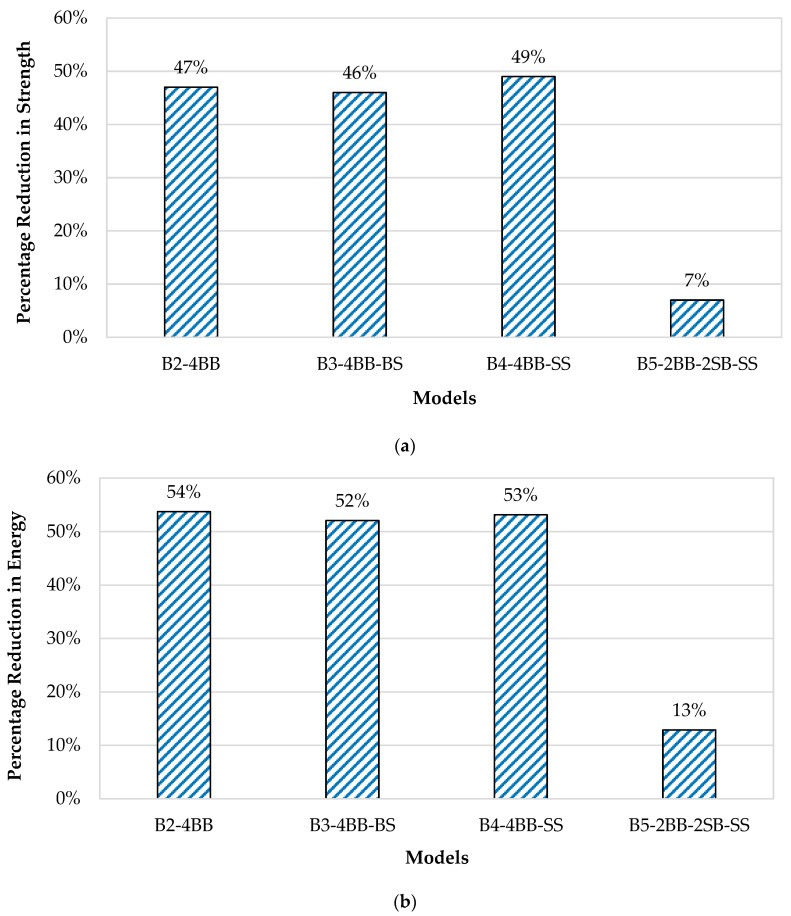
(**a**) Comparison of strength reduction percentage for all beams in comparison to control beam (B1-4SB-SS); (**b**) comparison of energy reduction of all models with respect to B1-4SB-SS.

Load–deflection curves of all the models were obtained from the analysis and are shown in Figure 12a. Figure 13a displays the reduction in the strength percentage for all the beams in comparison to the control beam (B1-4SB-SS). The load–deflection curve exhibited by B1-4SB-SS served as the reference curve. After a thorough analysis of the load–deflection curve for B2-4BB, it is apparent that its ultimate strength was 47% lower than that of B1-4SB-SS. Likewise, the load–deflection curves for both B3-4BB-BS and B4-4BB-SS clearly indicated reductions in the ultimate strength by 46% and 49%, respectively, when compared to B1-4SB-SS. Furthermore, this analysis revealed that there were negligible alterations in the beam’s strength solely by substituting bamboo stirrups with steel ones. It is crucial to emphasize the significant role that stirrups played in ensuring the effective anchoring of the primary reinforcement bars and aiding in the resistance against diagonal shear cracks. In addition, the load–deflection curve of B5-2BB-2SB-SS highlighted a marginal decrease in the ultimate strength, approximately 7%, in comparison to B1-4SB-SS. Notably, the hybrid BRC beam, represented by B5-2BB-2SB-SS, demonstrated considerable strength enhancements of approximately 40%, 39%, and 42% when compared with B2-4BB, B3-4BB-BS, and B4-4BB-SS, respectively.

The numerical model was further evaluated by comparing the energy absorption and failure pattern of all the beams. The calculation of the energy absorption for all the models was conducted by examining the area beneath the load–deflection curve, as displayed in Figure 12b. Figure 13b presents a comparative analysis of the decrease in the energy absorption for all the models in relation to the control model (B1-4SB-SS). The energy absorption for B1-4SB-SS was 2134.3 kN-mm, however, it was 988.13 kN-mm for B2-4BB, which was 54% less than that of B1-4SB-SS. Similarly, the energy absorption values for B3-4BB-BS and B4-4BB-SS were 1023.7 kN-mm and 1000.7 kN-mm, respectively. B5-2BB-2SB-SS had an energy absorption of 1859.27 kN-mm, which was about 13% less than that of B1-4SB-SS.

**Figure 14:**
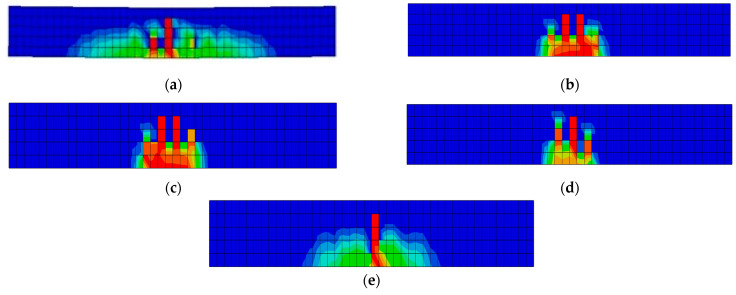
Tensile damage in beams: (**a**) B1-4SB-SS (control); (**b**) B2-4BB; (**c**) B3-4BB-BS; (**d**) B4-4BB-SS; (**e**) B5-2BB-2SB-SS.

Regarding the distribution of the tensile damage, B1-4SB-SS experienced damage spread over a larger area, as depicted in Figure 14a. Conversely, B2-4BB, B3-4BB-BS, and B4-4BB-SS illustrated the tensile damage that was distributed over considerably smaller areas when compared to B1-4SB-SS. These distributions are displayed in Figure 14b–d, respectively. Interestingly, the tensile damage for B5-2BB-2SB-SS was very similar to B1-4SB-SS, as shown in Figure 14e. These findings suggest that B5-2BB-2SB-SS could be a suitable replacement for B1-4SB-SS, offering an economically and environmentally friendly alternative.

## 5. Conclusions

In this study, a comprehensive exploration was conducted on the feasibility of replacing traditional steel reinforcement with bamboo in concrete beams utilizing validated numerical models. Also, a GA-based predictive model was developed to estimate the flexural strength of the BRC beams. Based on the analytical outcomes and the insights gained through this research, the following key findings and implications can effectively be addressed:

- A hybrid beam configuration with 50% steel and 50% bamboo reinforcements in the tension region can achieve a competitive ultimate strength, with only the marginal 7% reduction compared to a conventional SRC beam. This proportion of bamboo replacement for steel in beam construction holds great promise for significantly reducing the reliance on steel resources in the building and construction industry.
- The proposed hybrid bamboo–steel beams revealed comparable serviceability performance while requiring less reinforcement. The energy absorption of the BRC and SRC beams proved to be quite similar, with the minimal difference of only 13%. This suggests that the BRC beams can meet the required performance standards while being an environmentally sustainable and cost-effective alternative.
- The developed GEP-based predictive model proved to be a robust tool for estimating the flexural strength of the BRC beams. By incorporating key parameters such as the cross-sectional area of concrete, area of reinforcements, concrete compressive strength, and span and depth of the beams, this model achieved an impressive 97% accuracy. This highlights its potential as a valuable tool for engineers and designers in the building and construction industry.

While this study provides valuable insights into the potential of bamboo as a reinforcement material, there remains a need for further investigations. Future research should explore the use of bamboo reinforcement in conjunction with steel in various concrete components to better understand its versatility and applicability in real-world construction scenarios. Additionally, the incorporation of bamboo fibers as a means to enhance the strength of BRC beams warrants exploration as a valuable avenue for future research.

## Figures and Tables

**Table 1:** Mechanical and material properties [55].

Material	Density (kg/m^3^)	Young’s Modulus (MPa)	Poisson’s Ratio	Compressive Strength (MPa)	Tensile Strength (MPa)
Bamboo	700	18,475	0.20	90	90
Steel	7850	200,000	0.30	400	400
Concrete	2300	33,600	0.15	31.31	3.13

**Table 2:** CDP parameters.

Plasticity Parameter	Notation	Value
Dilation angle	*ψ*	40
Eccentricity	*ϵ*	0.1
Stress ratio	$\frac{\sigma_{b0}}{\sigma_{c0}}$	1.16
Shape factor	*K*	0.66
Viscosity	*μ*	0

**Table 3:** Description of beam models of this study.

Designation	Reinforcement Type	Description
B1-4SB-SS (Control)	Steel	Reference beam with steel bars and steel stirrups
B2-4BB	Bamboo	Beam only with bamboo bars
B3-4BB-BS	Bamboo	Beam with bamboo bars and bamboo stirrups
B4-4BB-SS	Bamboo and steel	Beam with bamboo bars and steel stirrups
B5-2BB-2SB-SS	Bamboo and steel	Beam with 50% steel bars, 50% bamboo bars, and steel stirrups

**Table 4:** Comparing experimental findings with FEM results.

Beam	*P_u_* (kN)		
	Experiment	FEM	Experiment/FEM
SRC	51	49.6	1.028
BRC	35	34.22	1.023

**Table 5:** Summary of critical influencing parameters.

Parameter	Range
Concrete compressive strength (*f’_c_*)	10–50 MPa
Span length (*L*)	100–140%
Area of reinforcement (*A_r_*)	2–8%
Area of cross-section (*A_c_*)	100–150%
Depth of beam (*D*)	200–350 mm

**Table 6:** Parameters for construction of model.

Function Set	+, −, /, x
Number of chromosomes	50
Head size	10
Number of genes	3
Linking function	Addition
One-point recombination	0.0027
Two-point recombination	0.0027
Constants per gene	10
Gene recombination	0.0027
Gene transposition	0.0027
Lower/upper bound of constants	−10/10

## Data Availability

The data are made available on request.
